# Economic consequences of caesarean section delivery: evidence from a household survey in Tanzania

**DOI:** 10.1186/s12913-021-07386-0

**Published:** 2021-12-29

**Authors:** Peter Binyaruka, Amani Thomas Mori

**Affiliations:** 1 Department of Health System, Impact Evaluation and Policy, Ifakara Health Institute, PO Box 78373, Dar es Salaam, Tanzania; 2 Chr. Michelsen Institute, PO Box 6033, N-5892 Bergen, Norway

**Keywords:** Caesarean section, Direct costs, Indirect costs, Obstetric care, Tanzania

## Abstract

**Background:**

Caesarean section (C-section) delivery is an important indicator of access to life-saving essential obstetric care. Yet, there is limited understanding of the costs of utilising C-section delivery care in sub-Saharan Africa. Thus, we estimated the direct and indirect patient cost of accessing C-section in Tanzania.

**Methods:**

Cross-sectional survey data of 2012 was used, which covered 3000 households from 11 districts in three regions. We interviewed women who had given births in the last 12 months before the survey to capture their experience of care. We used a regression model to estimate the effect of C-section on costs, while the degree of inequality on C-section coverage was assessed with a concentration index.

**Results:**

C-section increased the likelihood of paying for health care by 16% compared to normal delivery. The additional cost of C-section compared to normal delivery was 20 USD, but reduced to about 11 USD when restricted to public facilities. Women with C-section delivery spent an extra 2 days at the health facility compared to normal delivery, but this was reduced slightly to 1.9 days in public facilities. The distribution of C-section coverage was significantly in favour of wealthier than poorest women (CI = 0.2052, *p* < 0.01), and this pro-rich pattern was consistent in rural districts but with unclear pattern in urban districts.

**Conclusions:**

C-section is a life-saving intervention but is associated with significant economic burden especially among the poor families. More health resources are needed for provision of free maternal care, reduce inequality in access and improve birth outcomes in Tanzania.

## Background

Many country’s health systems are committed to achieve universal health coverage (UHC) goal to ensure healthy lives for all, under the Sustainable Development Goal three [1]. The UHC goal ensures that everyone has access to good quality health care without incurring financial hardship due to health care payment [2]. However, out-of-pocket (OOP) payment is a major means of financing healthcare in low- and middle-income countries (LMICs), which expose high proportion of households into poverty due to catastrophic healthcare expenditure [2–4]. Financing health care through direct OOP payments or user fees is typically regressive –i.e., the poorest are paying a relatively higher share of their income than their counterparts [3, 5–7]. The cost burden of health care also includes the indirect costs (e.g., time/ opportunity costs) which account for the loss in productivity due to medical illness [4, 8] and can equally limit access to health care as for direct costs [9–11].

The chances of incurring economic costs depend on many factors like the opportunity of accessing care, the existence of health financing policies which can ensure financial risk protection, and the nature of the illness. In terms of access, evidence shows that the direct financial costs are major barriers to access health care, which disincentive people to seek care [11–13]. Moreover, the health financing system which relies on OOP payments as opposed to prepayment mechanisms (e.g., tax funding and health insurance) often expose a large population, especially the low-income populations, into financial hardship due to medical spending [6, 14]. Similarly, the user fee exemption and waiver policy for the poor and vulnerable groups can potentially offer financial protection [15], but these policies are weakly enforced in many settings due to inadequate budgetary allocation to the health sector [16–18] as well as difficulties to identify the eligible clients [19, 20]. Lastly, the nature of illness also influences patients to incur economic costs –e.g., maternal obstetric complications are often unplanned and associated with large financial costs and productivity loss due to hospitalisation [8, 21, 22].

Several studies have examined the economic consequences of illness and associated coping mechanisms in LMICs [4, 23, 24], but not much has been reported in sub-Saharan Africa. For instance, the economic consequences of maternal illness/ obstetric care have been documented in Bangladesh [8, 22, 25–27], Nepal [28], Pakistan [29], and Argentina [30]. They generally found that maternal obstetric care (including C-section) was associated with higher direct costs and productivity loss. Knowledge about the economic costs of obstetric care remains limited in sub-Saharan Africa [31], despite the over-reliance on OOP payments for health services. Only a few studies in sub-Saharan Africa (e.g., [7, 17, 32–34]) shows that households are still incurring substantial direct costs for C-section delivery care irrespective of the exemption policy in those settings. These studies, however, hardly examined the indirect/ time costs of C-section due to hospitalisation, incremental costs of C-section after adjusting for covariates, and associated equity in C-section coverage.

In this paper, we provided the evidence on the estimates of both direct (OOP payments) and indirect cost (hospitalisation time) of C-section and normal delivery care in the context of free maternal health care in Tanzania. We also estimated incremental costs of C-section compared to normal delivery care and assessed equity in C-section coverage. Estimating costs of C-section in LMICs is preferred because C-section is an important indicator of access to life-saving essential obstetric care [35], and the burden of global maternal deaths is disproportionately higher in LMICs [36]. Both direct and time-related costs should be monitored to better understand the holistic view of cost burdens and barriers, especially in LMICs. Our findings are therefore relevant to inform policy discussions concerning health care financing for improved maternal and neonatal health outcomes and inform the discussions on financial protection towards achieving UHC.

## Methods

### Study setting

This study was conducted in three regions of Pwani, Morogoro and Lindi, out of 31 regions in Tanzania. These regions were considered because we used data from the evaluation of pay-for-performance (P4P) programme which was implemented in Pwani region, and used four districts from Morogoro and Lindi region for comparison [37, 38]. The population of Pwani region is just above a million, over two million in Morogoro region, and less than a million in Lindi region [39]. All seven districts in Pwani region were included in this study, and three districts from Morogoro and one district from Lindi were sampled. The country has made a lot of progress on child survival, but with little improvement in maternal health, which stands at 556 deaths per 100,000 live births [40, 41]. Access to one antenatal care (ANC) visit is almost universal, but relatively low coverage of institutional delivery (63%) and postnatal care (PNC) (33%) [40]. This reflects a marked imbalance along the continuum of maternal health care as reported elsewhere [42–44]. In 2016, the rate of C-section deliveries was 6%, and more likely among women who were wealthier, educated and residing in urban areas [40]. More than 70% of health facilities in Tanzania are publicly owned and are organised in a hierarchical administrative structure (i.e., dispensaries and health centres providing primary health care services, while up the rank there are district, regional, national and specialized hospitals that provide referral care).

The Tanzanian health financing system has multiple funding sources. In 2015/16 for example, the share of financing source to health care included general taxation (34%), donor support (36%), out-of-pocket payments (22%), and health insurance contributions (8%) [45]. In 2018/2019, about 9% of total government expenditure was allocated for health, which is below the Abuja declaration target of 15% [46]. About 34% of Tanzanians are covered by health insurance –i.e. 8% as public servants mainly through National Health Insurance Fund (NHIF), 25% as informal workers through Community Health Fund (CHF), and 1% from private insurance [46]. The coverage of health insurance is still low especially among the poor and informal workers. Tanzania has the exemption and waiver policies to protect the poor and vulnerable groups (e.g., pregnant women, children, and elders) [15, 47], but the enforcement of these policies has been weak such that exempted patients are still paying OOP [16, 48].

### Data sources

Data for this study were collected as part of a larger project evaluating the impact of a P4P programme in Pwani region [37, 38]. We specifically used the baseline data of the evaluation study. The survey was done in all seven districts in Pwani region, three  districts in Morogoro, and one district in Lindi region. The criteria for selecting the districts are presented elsewhere [38]. A cross-sectional survey of 3000 households was carried out in all 11 districts. Eligible household had a woman aged (15–49 years) who gave birth 12 months before the survey [38]. We included 150 facilities (12 hospitals, 32 health centres and 106 dispensaries) as the primary sampling unit, such that a random sample of 20 eligible households were drawn from each health facility’s catchment population [38]. The household survey was carried out by trained enumerators between January and February 2012. The structured questionnaire was administered to the household head and the eligible woman. The survey tool was designed to capture household background characteristics, and women’s experience of care specific for maternal and child health services, including associated direct and indirect costs. The survey tool was translated in Swahili and all the interviews were conducted in Swahili. A tool was pre-tested for consistency, relevance, and clarity before the actual survey.

### Costs of health care

We measured both direct (OOP payments) and indirect costs (hospitalisation time) through the survey tool. Irrespective of the fee exemption policy or insurance coverage, women were asked whether she or anyone else paid for delivery care services received at a health facility. Those who acknowledged paying were asked the following questions: *‘How much did you pay in total excluding the cost of transport to reach the facility? (In Tanzanian shillings, TZS)*’ and ‘*How long did you spend in the health facility from time of arrival to time of departure? (in hours)’*. Thus, through these two questions, we estimated direct and indirect costs, respectively. The direct costs excluded transport costs and were often paid for a consultation fee, drugs, medical supplies, laboratory tests, inpatient costs, informal payment/ gift to the health worker, or operation costs. Time costs were reported in terms of hours and then converted into days; while direct costs were reported in a local currency, Tanzanian shilling (TZS), and then converted into US dollar (USD) using the approximate exchange rate during the survey in 2012 (1 USD equal 1600 TZS).

### Statistical analysis

We first performed a descriptive analysis of the costs and background characteristics of respondents by mode of childbirth. Women were classified into two main groups: (i) those with normal delivery, and (ii) those with C-section delivery. The differences in costs and background characteristics by mode of childbirth were computed and tested whether those differences were significantly different from zero by using t-tests. To estimate the effect of C-section on costs, we applied a series of regression models by accounting for various households’ and women characteristics. The following regression model was estimated:

1 $${Y}_i={\beta}_0+{\beta}_1{D}_i+{\beta}_2{X}_i+{\varepsilon}_i$$

where *Y*_*it*_ is the cost incurred by individual *i* and *D*_*i*_ is an indicator dummy for woman delivered by C-section. We controlled for individual and household-level covariates *X*_*i*_ (age, marital status, religion, parity, education level, occupation, insurance status, household size, household wealth status, and place of residence). The error term is *ε*_*i*_. We clustered the standard errors at the facility level, or facility catchment area, to account for serial correlation of *ε*_*i*_ at the facility level. The effect of C-section childbirth on costs is given by *β*_1_. The reference group in this analysis is women who had a normal delivery. Our analysis was performed for all women with facility birth (85.8%) as well as for those who delivered in public facilities only (77.3%) (since fee-charging is typical in private facilities).

Given that cost data are typically skewed, with non-normal distribution, we also normalised our data by applying logarithm transformation [49]. We generated a variable using the following formula: In (1 + cost), in order to account for zero payments. Thus, we re-estimated our models using ordinary least-squares (OLS) for logged dependent variables –i.e., ln(*Y*_*i*_).

We further assessed the distribution of C-section coverage and costs across households’ socioeconomic status and place of residence (rural/urban). A wealth index was computed as a measure of household living standard. We used principal component analysis based on 42 items of household characteristics and asset ownership to generate a wealth score for each household (Appendix A1) [50, 51]. Households were then ranked according to the wealth index/ score and categorized into quintiles of equal size, with quintile 1 consisting of the poorest 20% households, while quintile 5 consisted of the least poor 20%. We presented our equity results in three aspects: a bar graph and concentration curve showing the distribution of C-section coverage across quintiles and then computed the corresponding concentration index. The concentration index is defined as twice the area between the 45-degree line of equality and the concentration curve, and it measures the degree of socioeconomic inequality in a variable of interest [52]. A concentration curve plots the cumulative share of C-section coverage (Y-axis) against the cumulative share of the households ranked by socioeconomic status (X-axis). A dominance test was performed to assess whether the concentration curve is statistically different from the line of equality [52]. The concentration curve is then summarised by a concentration index, which ranges between [− 1 and + 1], whereby zero indicate equality across socioeconomic subgroups, while negative and positive values indicate pro-poor and pro-rich access to C-section delivery care, respectively [52]. We also tested whether a concentration index was significantly different from zero. All analyses were performed using STATA version 16.

## Results

### Descriptive statistics

Table 1 presents a description of the data by mode of delivery. The response rate was almost 96% (*n* = 2874) out of 3000 eligible women/ households. Out of 2874 women, 86% (*n* = 2466) had facility-based delivery care, 78% (*n* = 2229) had normal delivery and 8% (*n* = 237) had C-section delivery (7.5% of C-section were in public facilities only).

**Table 1:** Descriptive statistics by mode of delivery

Variable	All (1)	**Normal delivery** (2)	**C-section delivery** (3)	Difference (3)–(2)
**Panel A: Outcomes**	N = 2874	*N* = 2874	N = 2874	
Gave birth in a facility (%)	85.8 (N = 2466)	77.6 (N = 2229)	8.3 (N = 237)	
Probability of paying for facility birth (%)	20.5	19.1	34.4	0.153***
Amount paid for facility birth in USD, mean [sd]	3.8 [23.9]	1.9 [7.3]	22.7 [73.1]	20.8***
Hours of hospitalisation at facility, mean [sd]	29.5 [43.6]	24.6 [34.4]	77.2 [81.1]	52.6***
Days of hospitalisation at facility, mean [sd]	1.2 [1.8]	1.0 [1.4]	3.2 [3.4]	2.2***
Gave birth in a public facility (%) (N = 2874)	77.3 (*N* = 2221)	69.8 (*N* = 2005)	7.5 (*N* = 216)	
Probability of paying for public facility birth (%)	14.1	12.5	29.7	0.169***
Amount paid in public facility in USD, mean [sd]	2.0 [13.5]	0.9 [5.9]	11.9 [38.8]	11.0***
Hours of hospitalisation in public facility, mean [sd]	29.3 [43.1]	24.8 [34.3]	73.5 [80.05]	48.7***
Days of hospitalisation, mean [sd]	1.2 [1.8]	1.0 [1.4]	3.1 [3.3]	2.1***
**Panel B: Covariates**	(*N* = 2412)	(*N* = 2191)	(*N* = 221)	
Age of a woman in years [sd]	26.3 [6.6]	26.3 [6.6]	26.3 [6.9]	−0.017
Married woman (%)	0.669	0.669	0.674	0.005
Muslim woman (%)	0.764	0.766	0.738	−0.028
Parity/number of births, mean [sd]	2.6 [1.7]	2.6 [1.7]	2.2 [1.6]	−0.4***
Educated with at least some primary education (%)	0.821	0.816	0.859	0.043
Farmer (%)	0.478	0.494	0.321	−0.173***
Insured woman (%)	0.087	0.084	0.112	0.028
Household size, mean [sd]	4.7 [1.8]	4.8 [1.8]	4.5 [1.9]	−0.3*
Wealth quintile 1 (%) (poorest)	0.197	0.204	0.127	−0.077***
Wealth quintile 2 (%)	0.189	0.189	0.190	0.001
Wealth quintile 3 (%)	0.193	0.198	0.131	−0.067**
Wealth quintile 4 (%)	0.204	0.204	0.195	−0.009
Wealth quintile 5 (%) (least poor)	0.218	0.203	0.357	0.154***
Household in rural district (%)	0.809	0.817	0.724	−0.093**

Notes: sd = standard deviation; * p < 0.10, ** p < 0.05, *** p < 0.01

Sampled women were predominantly married, Muslim, educated at least with primary education, farmers, uninsured, and residing in rural district councils. On average, women were aged 26 years old, with almost 3 births, and residing in households with 5 family members. The two groups of women based on the mode of delivery were similar in terms of age, marital status, religion, education, and health insurance status.

Delivery at the health facility was associated with a 20% likelihood of incurring OOP payments. However, as expected, the likelihood of paying OOP almost doubled for C-section compared to normal delivery, i.e., 19% versus 34%. The average amount paid for C-section was almost 12 times that of normal deliveries, while hospitalisation time was 3 times that of normal deliveries. The pattern on cost comparison and hospitalisation time between modes of childbirth did not change even when the analysis was restricted to public facilities only.

### Effect of C-section delivery on direct costs

Table 2 shows the incremental chances of paying and the corresponding amount incurred by women who delivered by C-section after accounting for covariates. The C-section delivery care was positively associated with high chances of paying for health care (16 percentage point) and around 18 percentage point in public facilities only. In terms of the odds ratio (data not shown), the likelihood of paying for C-section was higher (OR = 2.43, 95% CI: 1.75–3.35) than normal delivery, and similarly when restricting to public facilities only (OR = 3.19, 95% CI: 2.23–4.57). The incremental cost of giving birth by C-section, compared to normal delivery, was 20 USD on average and almost 11 USD in public facilities only.

**Table 2:** Effect of C-section delivery on delivery care costs

Dependent variable	Probability of paying		Amount paid (USD)		Log of the amount paid (USD)	
	All	Public facilities	All	Public facilities	All	Public facilities
C-section delivery	0.164*** (0.034)	0.179*** (0.034)	20.47*** (5.042)	10.59*** (2.668)	0.771*** (0.130)	0.671*** (0.112)
Mean of dep. Variable	0.205	0.141	3.768	2.011	0.464	0.291
R-squared	0.029	0.035	0.070	0.071	0.063	0.074
No. of observations	2412	2173	2412	2173	2412	2173

Notes: Robust standard errors in parentheses; The reference group is women who delivered in a health facility with a normal delivery; Adjusted covariates include age, marital status, religion, parity, education level, occupation, insurance status, household size, household wealth status, and place of residence; * *p* < 0.10, ** *p* < 0.05, *** *p* < 0.01

### Effects of C-section delivery on indirect costs

The effects of C-section on productivity loss after accounting for covariates were presented in Table 3. We found a significant reduction in labour supply/ productivity after C-section delivery. On average, women who delivered by C-section spent 2 days or 51 h more for hospitalisation compared to women with normal delivery (Table 3). The productivity loss slightly reduced to 1.9 days and 47 h when restricting to public facilities only.

**Table 3:** Effect of C-section delivery on labour supply/ productivity loss

Dependent variable	Hours of hospitalisation		Log of hours of hospitalisation		Days of hospitalisation		Log of days of hospitalisation	
	All	Public facilities	All	Public facilities	All	Public facilities	All	Public facilities
C-section delivery	50.99*** (5.629)	46.91*** (5.859)	0.859*** (0.114)	0.782*** (0.119)	2.125*** (0.235)	1.955*** (0.244)	0.540*** (0.059)	0.496*** (0.062)
Mean of dep. Variable	29.469	29.318	2.758	2.755	1.23	1.221	0.626	0.625
R-squared	0.135	0.122	0.074	0.065	0.135	0.122	0.115	0.101
No. of observations	2412	2173	2412	2173	2412	2173	2412	2173

Notes: Robust standard errors in parentheses; The reference group is women who delivered in a health facility with a normal delivery; Adjusted covariates include age, marital status, religion, parity, education level, occupation, insurance status, household size, household wealth status, and place of residence; * *p* < 0.10, ** *p* < 0.05, *** *p* < 0.01

### Equity in C-section utilisation 

**Fig. 1:**
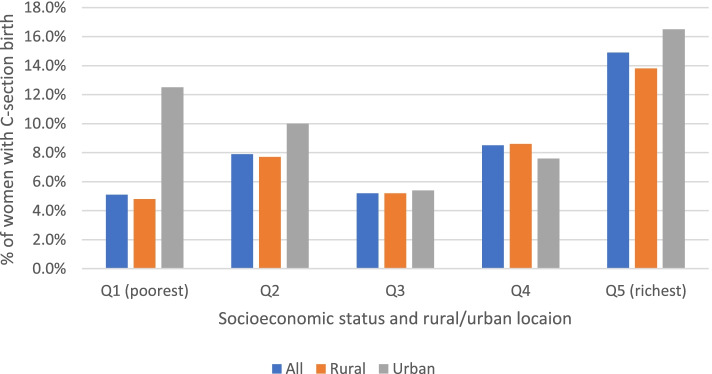
Distribution C-section utilisation by wealth quintiles and location

**Fig. 2:**
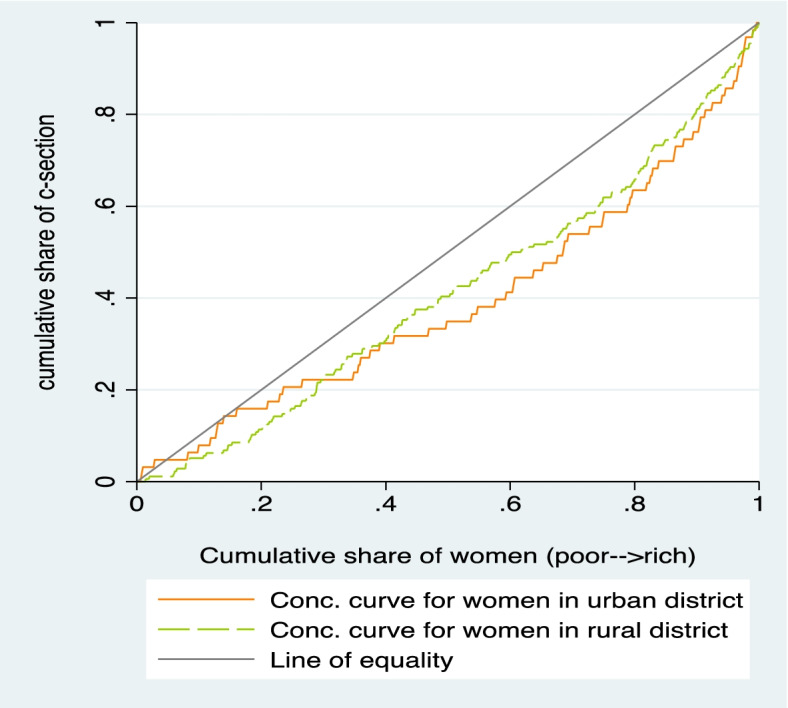
Concentration curves of the distribution of C-section coverage

As indicated in Fig. 1, the overall coverage of C-section delivery was lowest among women from the poorest households (5.1%) compared to those from the least poor households (14.9%). The corresponding concentration index was positive (CI = 0.2052, *p*-value< 0.01). All these indicates a pro-rich coverage in C-section care. However, the costs or average amount paid for delivery care were significantly higher among the richest than the poorest women (data not shown). In terms of location, the C-section coverage was higher in urban (12.1%) than in rural districts (7.5%). We further assessed the inequality by wealth status within urban and rural setting separately. While a similar pattern of pro-rich was observed for C-section coverage by socioeconomic quintiles among rural women, there was unclear pattern among women in urban districts since middle groups had the lowest coverage (Figs. 1 and 2). In terms of concentration curves, both curves for rural and urban were below the line of equality indicating a pro-rich utilisation of C-section care. However, the pro-rich inequality in rural areas was significantly different from zero (CI = 0.1786, *p* < 0.01) than that of urban setting with a borderline significance (CI = 0.2143, *p* < 0.10). Since the two concentration curves crosses each other, a dominance test was performed to confirm whether the two curves were significantly different. The test confirmed that there was no evidence of dominance, indicating lack of enough evidence that one curve dominating the other. However, the curves in the richest quintiles clearly indicated relatively higher pro-rich inequality in urban than rural, as the curve in urban lies above that of rural. In contrast, the curves in the lowest quintiles were reversed such that the urban curves almost touched the line of equality and lies above the curve for rural, indicating continued pro-rich coverage in rural with unclear pattern for urban.

## Discussion

This study found that C-section was significantly associated with higher chances of paying for health care, and had relatively higher direct and indirect costs to patients compared to normal delivery. Women from wealthier and urban households were more likely to access and receive C-section delivery and paid more direct costs on average than their counterpart women.

The prevalence of C-section deliveries was slightly higher (8.3%) than the national average of 6% reported in the 2015/16 Tanzania Demographic and Health Survey [40] and regional average of 7.3% for Africa [53]. The low coverage rate of C-section is typical in developing countries when compared to high-income countries [54, 55]. One of the reasons for low uptake of C-section in developing countries is the inadequate infrastructural and human resource capacity to offer emergency and surgical care [56–58]. For instance, only 19–50% of hospitals in sub-Saharan Africa can provide 24-h emergency care. However, as countries reform their health systems and improve health care service utilisation [59–61], one would expect the C-section rates would increase over time.

The result of higher patient costs for C-section than normal delivery is consistent with the previous pattern reported elsewhere [7, 8, 17, 22, 25, 31, 33, 62, 63], although the incremental cost of 20 USD that we found was relatively lower. In comparison, the incremental cost for maternal complications/ C-section was 13.6 USD in Mali [7], 55.9 USD in Democratic Republic of Congo [33], and varied by time and measurement in Bangladesh including 86 USD per C-section birth [27], 34 USD per month [8] and around 269 USD from childbirth to six months postpartum [22]. A few studies in a recent review in sub-Saharan Africa reported the costs of C-section delivery ranging from 55.8–377.3 USD [31]. In Pakistan, postpartum mother after C-section incurred 204 USD (79 USD for normal delivery) as total direct and indirect cost including transport and food [29]. These costs incurred by patients and/or relatives suggest that accessing essential obstetric care including C-section can reduce household resources significantly [8, 22] and can reinforce catastrophic health spending [7, 64, 65].

Our study also revealed that C-section delivery was associated with much higher loss in productivity compared to normal delivery. A similar finding, though for maternal complications, was reported in Bangladesh [8, 22] and Ghana [34]. While women in Tanzania were hospitalised for an average of 2 days after C-section, women with maternal complications in Bangladesh lost 2 to 3 days after childbirth [8]. Another study in Bangladesh valued higher productivity loss between 30.1–33.1 USD for severe and less-severe complication than 14.1 USD for normal delivery [25]. Similarly, Ghanaian women with maternal complications spent 3 days on average (2 days median) for hospitalisation, while average productivity loss was estimated to be 8.92 USD [34].

The assessment of equity in health care benefits and payments is an important approach to monitor progress towards UHC [6, 66]. Equity is particularly needed to ensure that households receive health benefits according to their health care need and contributes to the health care according to their ability-to-pay [5, 66]. The available evidence is often in contrast to the above equity principle for UHC [6]. For instance, this study found that C-section delivery was more likely to be accessed by wealthier and urban women, yet inaccessible by their counterparts that may have the greatest health care need. The Tanzania Demographic and Health Survey also reports higher rates of C-section deliveries among the wealthier, educated and urban residing women [40]. This ‘socioeconomic gradient’ in utilising C-section have also been reported elsewhere [54, 55, 67–69]. In terms of equity in health care payment, however, the burden of direct payments was significantly higher among the richest as they are more likely to access C-section care than the poorest women. Consistently, the poorest typically spend less on treatment than other income groups due to lack of access, inability to pay, greater use of public services [23].

Fair and timely access to essential life-saving interventions is needed to reduce morbidity and mortality rates globally. For instance, to reduce maternal deaths may need fair and timely access to basic and comprehensive emergency obstetric care [70]. Consequently, many LMICs decided to offer ‘free maternity services’ or implement a user fee exemption policy to reduce the financial barriers [71–75]. Yet, a large body of evidence in these settings shows women are still paying for exempted services and facing financial barrier [16–18, 27, 65, 71, 76, 77]. One reason for such weak enforcement of free/ exemption policy is existing disruptions in health systems [71] including an inadequate budget allocation to the health sector [16–18] as well as difficulties to identify the eligible clients [19, 20]; which altogether undermines the effectiveness of the policy [78–82].

Our findings have important policy implications. Despite the efforts to reduce the direct medical costs by offering ‘free maternity services’ in many settings including Tanzania (or offering fee exemption for C-section specific in some settings), evidence shows that people are still paying OOP for exempted/ free services. This indicates weak enforcement of the policy and eventually affecting the effort to offer financial protection for UHC. The lack of formal waivers/exemption directives to health workers are considered to undermines the legal basis for effective implementation of free maternity services. It is also well documented that patients may incur costs in relation to informal payment/ gift to the health worker, particularly when supportive supervision is ineffective, health workers having poor working conditions (e.g., drug stockout, inadequate staffing level) and are not renumerated on timely manner (e.g., salaries, benefits) [83]. Access to C-section care is also in favour of the better-off, which reflects the low affordability among the poorest population [7, 64]. It further implies that some women, especially the worse-off, are deterred to access life-saving interventions or losing their lives as they cannot afford C-section delivery care [7, 84]. It is even life-threatening concerning the unaffordability of life-saving interventions such as C-section since this care increases the chance of rehospitalisation [85]. Since C-section is an emergence and life-saving procedure for the mother and the baby, fair and timely access irrespective of women characteristics is necessary. Countries should therefore ensure timely access to effective and affordable basic and comprehensive emergency obstetric care to reduce maternal deaths [35, 86]. Efforts are also needed to improve access to surgical services especially in sub-Saharan Africa [87]. To reduce financial risks especially among the poor in the move to UHC, adequate funding to health facilities through prepayment mechanisms and strong enforcement of the exemption policy or user fee removal would help [2, 66]. Further research is needed to deeply understand the coping mechanisms and main drivers of paying for exempted services in Tanzania.

This study has the following limitations. First, we were unable to incorporate transport costs to access care due to data availability but its significant contribution to catastrophic health spending is well documented [4]. This data was lacking because the main evaluation study was not designed to accommodate this information. Otherwise, the study may have underestimated the actual patient costs associated with seeking C-section services. Second, while the assessment of the affordability of costs regarding C-section is important [7], we did not get data on household income/ expenditure to reflect a household’s ability to pay. Third, we were unable to identify the coping strategies to finance delivery care because of the limited data available. Fourth, the productivity loss was not quantified in monetary values, because of unreliable income or wage rate data for the rural and urban population. Fifth, the information about women’s medical conditions prior to C-section was not collected, while this information could be adjusted and used to explain the findings. Lastly, there is a possibility of a recall bias as we relied on recall data for costs incurred during childbirth in the last 12 months.

## Conclusions

Despite the presence of exemption policy for maternal health services in Tanzania, women accessing and utilising delivery care in health facilities are facing substantial direct and indirect costs, and significantly higher costs for obstetric or C-section delivery care. The exemption or user fee removal policy is an important arrangement to address the financial barriers to access essential obstetric care like C-section, but not a sufficient approach to ensure financial protection in poor-resource settings. To achieve the intended policy goal, countries should ensure strong enforcement of exemptions through reduced health system disruptions and timely reimbursement of resources/ budget disbursements to cover the exempted costs. These efforts are needed not only to improve access to obstetric care but also to ensure financial protection for UHC as well as reduce maternal deaths.

## Abbreviations

ANC: Antenatal Care
C-section: Caesarean Section
CHF: Community Health Fund
CI: Concentration Index
LMICs: Low and Middle-Income Countries
NHIF: National Health Insurance Fund
OLS: Ordinary Least Square
OOP: Out-Of-Pocket
OR: Odds Ratio
PNC: Postnatal Care
TZS: Tanzanian Shillings
UHC: Universal Health Coverage
USD: United States Dollar
